# Ubiquitination of ASCL1 mediates CD47 transcriptional activation of the AKT signaling pathway, and glycolysis promotes osteogenic differentiation of hBMSCs

**DOI:** 10.1007/s11626-023-00811-0

**Published:** 2023-10-02

**Authors:** Jimei Zhang, Ling Zhu, Jianping Zhou, Qunying Yu, Guangyuan Yang, Ke Zhao, Chaoli Luo, Jianguo Meng, Jing Liu, Xuming Yang

**Affiliations:** 1 Department of Gastroenterology, Chenggong Hospital, Yan an Hospital Affiliated to Kunming Medical University, Kunming, 650505 China; 2 Department of Orthopedics, Chenggong Hospital, Yan an Hospital Affiliated to Kunming Medical University, Kunming, 650505 China; 3 Department of Obstetrics, The Second Affiliated Hospital of Kunming Medical University, Kunming, 650101 China; 4 Kunming Medical University, Kunming, 650500 China; 5 Department of Orthopedics, Yunnan Pain Disease Hospital, Kunming, 650224 China; 6 Operating Room, Yunnan Pain Disease Hospital, Kunming, 650224 China; 7 Department of Orthopedics, Guangnan Hospital of Traditional Chinese Medicine, Yunnan Province, Guangnan, 663300 China; 8 Department of Orthopedics, Yan an Hospital Affiliated to Kunming Medical University, Kunming, 650055 China

**Keywords:** hBMSC osteogenic differentiation, Glycolysis, Ubiquitination, ASCL1, CD47

## Abstract

Bones are extremely dynamic organs that continually develop and remodel. This process involves changes in numerous gene expressions. hBMSC cells can promote osteogenic differentiation. The purpose of this study was to elucidate the mechanism by which ASCL1 promotes osteogenic differentiation in hBMSC cells while decreasing glycolysis. hBMSCs were induced to differentiate into osteoblasts. The ASCL1 expression level during hBMSC osteogenic differentiation was measured by RT‒qPCR, Western blotting, and immunofluorescence. The differentiation level of osteoblasts was observed after staining with ALP and alizarin red. ChIP-qPCR were used to determine the relationship between ASCL1 and CD47, and the expression of glycolysis-related proteins was detected. Overexpression of ASCL1 was used to determine its impact on osteogenic differentiation. si-USP8 was used to verify the ubiquitination of ASCL1-mediated CD47/AKT pathway’s impact on hBMSC glycolysis and osteogenic differentiation. The results showed that the expression of ASCL1 was upregulated after the induction of osteogenic differentiation in hBMSCs. From a functional perspective, knocking down USP8 can promote the ubiquitination of ASCL1, while the osteogenic differentiation ability of hBMSCs was improved after the overexpression of ASCL1, indicating that ASCL1 can promote the osteogenic differentiation of hBMSCs. In addition, USP8 regulates the ubiquitination level of ASCL1 and mediates CD47 transcriptional regulation of the AKT pathway to increase the glycolysis level of hBMSCs and cell osteogenic differentiation. USP8 ubiquitination regulates the level of ASCL1. In addition, ubiquitination of ASCL1 mediates CD47 transcription to activate the AKT signaling pathway and increase hBMSC glycolysis to promote osteogenic differentiation.

**Supplementary Information:**

The online version contains supplementary material available at 10.1007/s11626-023-00811-0.

## Introduction

Osteoporosis has become a disease that is detrimental to human health worldwide, and its prevalence is showing an increasing trend, especially among middle-aged and elderly women (Xiao *et al*. 2022; Kanis *et al*. 2019). One of the important factors leading to osteoporosis is the imbalance between bone formation and bone resorption during bone reconstruction, and the decline in osteogenic differentiation is the primary factor leading to the occurrence of osteoporosis (Wang *et al*. 2021). Current clinical treatments for osteoporosis include bisphosphonates, selective estrogen receptor modulators (SERMs), and hormone replacement therapy (HRT), which focus on the management of its symptoms (Wang *et al*. 2020). Despite significant progress in the discovery of osteoporosis medications, the currently available treatments are limited by their side effects and long-term safety (Palacios 2022). Therefore, the clinical need to develop more effective and curative approaches, through a better understanding of disease progression at the molecular level, remains.

Mesenchymal stem cells (MSCs) are adult stem cells with self-renewal capacity and multidirectional differentiation potential from sources such as bone marrow, adipose, placenta, umbilical cord, and dental pulp, with approximately 60% derived from bone marrow (Thanaskody *et al*. 2022). The properties of MSCs have been described by Dominici *et al*. (2006): plastic adherence under standard culture conditions, positivity for the surface receptors CD73, CD90, and CD105 and negativity for CD11b, CD14, CD19, CD34, CD45, CD79α, and human leukocyte antigen histocompatibility DR antigen (HLA⁃DR), and the ability to differentiate in vitro toward osteoblasts, adipocytes, and chondrocytes. Osteogenic differentiation is a key factor in bone regeneration, and human bone marrow mesenchymal stem cells (hBMSCs) have been identified as pleiotropic stromal cells with multispectral differentiation capacity and immunosuppressive properties (Yu *et al*. 2021). Increasing evidence suggests that these characteristics of hBMSCs are associated with the secretion of extracellular carriers, which are rich in functional components such as proteins and microRNAs (miRNAs) (Kuang *et al*. 2023). Currently, hBMSC-derived external vesicles (EVs) have become an effective tool for a variety of diseases with important features (Li *et al*. 2021). Nevertheless, little is known about the mechanisms of hBMSC action on osteogenesis, especially in the regulation of osteogenic differentiation. Therefore, it is particularly important to thoroughly explore the specific mechanisms of action of hBMSCs in the treatment of osteogenic differentiation.

The proliferation of MSCs is accompanied by oxygen glycolysis and oxidative phosphorylation (OXPHOS) (Mohammadalipour *et al*. 2020). Some stem cells and cancer cells undergo a process called aerobic glycolysis that is similar to the Warburg effect (Vander Heiden *et al*. 2009). The Warburg effect is a key link in bone formation. Bone formation takes glycolysis as the main metabolic mode, which consumes a large amount of glucose and produces lactic acid (Esen and Long 2014). However, different studies have shown that mesenchymal stem cells use OXPHOS more frequently under osteoblastic culture conditions (Pattappa *et al*. 2011). In addition, adipose-derived MSCs promote osteogenic differentiation by regulating cell energy metabolism and glycolytic ability (Bispo *et al*. 2022). It is worth noting that there is still controversy over whether glycolysis is involved during osteogenic differentiation and its regulatory molecular mechanisms.

Protein degradation has become a key factor in the occurrence of tumors. Ubiquitination is involved in the posttranslational modification process of protein degradation (Kitamura 2023), which affects the physiological activities of cells. Ubiquitination is a reversible modification, and ubiquitin-specific peptidase 8 (USP8) is a kind of deubiquitination enzyme that catalyzes the release of ubiquitin molecules (Gingerich *et al*. 2023). The deubiquitinating enzyme activity of USP8 can counteract the degradation of ubiquitinated protein substrates by the proteasome or lysosome (Pattappa *et al*. 2011). Achaete cut complex homolog like 1 (ASCL1) is a neuronal characteristic factor that induces the differentiation of a variety of cells (Long *et al*. 2023a). Previous studies have proven that the ASCL1 population bound by chromatin is related to the short chain of ubiquitin, while cytoplasmic ASCL1 contains a longer ubiquitin chain, and only cytoplasmic ubiquitination targets ASCL1 for destruction (Han *et al*. 2023). ASCL1 is abnormally overexpressed in USP8 mutations and wild-type tumors in Cushing syndrome (Gillotin *et al*. 2018). In addition, bone marrow stromal cells (BMSCs) transfected with Asc11 can differentiate into neuronal cells in vitro (Chen *et al*. 2022). However, to date, there has been no report on whether ubiquitination of ASCL1 mediates osteogenic differentiation of hBMSCs. 

CD47 is a complete glycoprotein cell receptor that is widely expressed at low levels in most healthy cells in the human body (Oldenborg 2013). Immunity, self-recognition, cell adhesion, and vascular tension depend on ligands or companion receptors associated with CD47 (Gwag *et al*. 2023). It is worth noting that the stable ASCL1 protein activates CD47 transcription and enhances cancer cell characteristics (Wang *et al*. 2022a). In addition, the PDK1 inhibitor BX795 mediates glycolysis in tumor cells by downregulating the PDK1/CD47/AKT signaling pathway (Pai *et al*. 2021). Liu *et al*. (2019) found that an increase in CD47 promotes the progression of human glioblastoma by regulating the PI3K/Akt signaling pathway. However, there is currently no evidence that ubiquitination of ASCL1 affects CD47/Akt signaling, glycolysis, and osteogenic differentiation of hBMSCs.

In this study, we aimed to explore the molecular mechanism by which USP8 participates in regulating the ubiquitination of ASCL1, affecting the transcriptional regulation of the AKT signaling pathway and glycolysis downstream of CD47, and mediating osteogenic differentiation in hBMSCs.

## Materials and methods

### Cell culture

Human bone marrow-derived BMSCs (hBMSCs) were purchased from Pricella (Wuhan, China) with product number CP-H166. Quality testing, hBMSC isolated by Pricella labs were identified by CD29 and CD90 immunofluorescence, the purity can be more than 90%, and do not contain HIV-1, HBV, HCV, mycoplasma, bacteria, yeast, and fungi. The cells were incubated in DMEM (ScienCell Research Laboratories, Inc., Carlsbad, CA) containing 10% FBS in a 5% CO_2_ and 37°C incubator. When hBMSCs were cultured to the fourth generation, cells were inoculated onto a 6-well plate (1 × 10^6^ cells/well). After 24 h, 2 mL of supplemented 10% FBS, 1% glutamine, 10 nM dexamethasone, 0.2 nM ascorbic acid, and 10 nM β-glycerophosphate osteogenic differentiation medium (OM) was added to each well. Cells were collected on days 0, 1, 7, and 14. 

### Induction of osteogenic differentiation in hBMSCs

The hBMSC cell suspension at a concentration of 4 × 10^6^/mL was taken and centrifuged at 500 × g for 15 min in a 15-mL plastic centrifuge tube to obtain microcell clusters. The microblocks were cultured with serum-free H-DMEM osteogenic induction medium containing 1 ng/mL TGF-β1, 10^−7^ mmol/L dexamethasone, 50 μg/mL vitamin C, 6.25 ng/L insulin, 6.25 μg/mL transferrin, and 1.25 μg/mL BSA. The culture medium was changed for the first time after 4 d and then every other day until the 14th day.

### Cell transfection

According to the manufacturer’s instructions, Lipofectamine 3000 (Thermo Fisher Scientific, Waltham, MA) was used to transfect NC, ASCL1, si-NC, and si-USP8 into hBMSCs. After 48 h, the cells were collected for drug treatment. The oe-NC, oe-ASCL1, si-NC, si-USP8, and oe-ASCL1 genes were designed and synthesized by Sangon Biotech (Shanghai, China). Stably transfected cells were selected with puromycin (3 mg/mL, Sigma-Aldrich, St. Louis, MO) for 5 d, and then, the cells were collected. The cells were collected, and a fixed dose (2.5 μM) was added according to the experimental requirements. The cells were treated with an AKT activator (SC79) for 24 h, and the detection indicators were evaluated.

### RT‒qPCR

Total RNA was extracted from cells using TRIzol reagent (Invitrogen, Carlsbad, CA). HiScript II Q Select RT Supermix (Novozan Biotechnology Co., Ltd. Nanjing, China) was used for reverse transcription. SYBR Green Master Mix (Novozan Biotechnology Co., Ltd.) was used to measure gene expression levels. All steps were completed according to the manufacturer’s instructions using the formula 2^− ΔΔ Ct^. The change was calculated in multiples and standardized to the internal reference gene GAPDH (mRNA). The primer sequences are shown in Table 1.

**Table 1.** Primer sequences

*Target*	*Sequence (F: forward primer, R: reversed primer)*
*ASCL1*	*F: 5′-CTCCTCTTGACATTTGCAGAGTG-3*′
	*R: 5′-GCAGTCTTGAAAGACTCGAGTGTG-3′*
*GAPDH*	*F: 5′-GCAACTAGGATGGTGTGGCT-3′*
	*R: 5′-TCCCATTCCCCAGCTCTCATA-3′*

### Western blot

RIPA lysis solution (Beyotime, Shanghai, China) was used to lyse cells. The concentration was measured by a BCA reaction kit. After quantitative analysis, the total protein was denatured in this study. An SDS‒PAGE gel was used for electrophoresis, and the electrophoresis apparatus (Bio-Rad, Hercules, CA) was adjusted to 120 V for electrophoresis. A PVDF membrane (Millipore, Boston, MA) was used for membrane transfer, and skim milk (Sigma) was used for blocking. Prediluted primary antibodies against ASCL1, bone morphogenetic protein 2 (BMP-2), Osterix (OSX), Osteocalcin (OCN), USP8, CD47, AKT, and GAPDH (the above antibody dilution ratios refer to the instruction manual of Abcam, Cambridge, UK) were added and incubated overnight. The next day, goat anti-mouse antibody (1:2000; Abcam) or goat anti-rabbit antibody was incubated for 1 h with slow shaking at 25°C. ECL chemiluminescence solution was used for development, a chemiluminescence instrument was used for exposure and observation, and ImageJ was used for protein band analysis.

### Immunofluorescence

Immunofluorescence analysis of hBMSCs was performed. The cells were fixed with 4% paraformaldehyde (PFA) and washed three times with PBS containing 0.1% Triton X-100. After sealing, the corresponding first antibody ASCL1 (ab211327, 1/100) was incubated overnight at 4°C. The corresponding fluorescently labeled secondary antibody was added for 60 min and rinsed twice in PBS. Cover the slide with a vector field installation medium containing the nuclear staining agent DAPI (Vector Laboratories, San Francisco, CA). The specimens were analyzed under a Leica SP5 confocal microscope. ImageJ was used to analyze nuclear localization, including automatic detection of nuclei in DAPI channels and obtaining DAPI staining and fluorescence-labeled images.

### Cell Counting Kit-8 (CCK-8)

hBMSCs were inoculated into 96-well plates at 1 × 10^4^ mL and incubated for 0, 7, and 14 d. At the end of each time point, 10 μL CCK-8 solution (Shanghai Yuanmu Biotechnology Co., Ltd. Shanghai, China) was used and then incubated at 37°C for 4 h. Cell proliferation was evaluated using an enzyme-linked immunosorbent assay at 450 nm.

### ALP staining

ALP activity was measured using an ALP assay kit (Nanjing Jiancheng Bioengineering Institute, Nanjing, China). Cells cultured in 6-well plates were taken, washed with PBS, and fixed by adding ALP fixative according to the manufacturer’s instructions. Subsequently, the cells were washed twice with PBS, while the ALP staining solution was added and incubated at 37°C for 4 h. Images were acquired by photographing under an Olympus BX-51 fluorescence microscope (Olympus Corp, Tokyo, Japan).

### Alizarin red staining

The level of mineralization was assessed by alizarin red staining. hBMSCs, after osteogenic induction, were washed with PBS and fixed in formaldehyde solution with a mass fraction of 10% at room temperature; after 15 min, they were washed with water, and a 0.1% alizarin red-Tris HCl (pH 8.3) solution was added at room temperature. The cells were gently incubated with shaking for 30 min, and the cells were incubated for 30 min with the criteria of red‒orange nodules and clear borders. Then, the cells were inverted in an inverted microscope. The formation of mineralized nodules was observed under an inverted microscope.

### Co-IP

Inoculate the treated cells onto a six well plate (5 × 10^6^ cells/well), add lysis buffer, probe with USP8 labeled with flag or ASCL1 labeled with hemagglutinin (HA) at 4°C for 3 h, centrifugate 300 × g for 10 min, add 40 µL sample buffer, and analyze immunoprecipitation protein.

### ChIP‒qPCR

ChiP qPCR was performed according to Wang *et al*. (2018). After ultrasonic chromatin was precleared with ChiP grade protein A/G and agarose for 1 h, 20 mL of hBMSC culture was harvested. The cells were harvested and fixed and washed with PBS. Then, 200 mg of cells was collected by ultrasound treatment on ice in 4 mL of lysis buffer, and chromosome DNA was fragmented into 500 ± 2 bp fragments. The preclarified supernatant containing a total of 5 mg of protein was mixed with 4 μL of the GlnR polyclonal antibody and incubated overnight at 50°C. At 4°C, 4 μL was incubated with protein G agarose (Thermo Fisher Scientific) for 1 h. After reverse cross-linking purification, high-quality ChIP DNA was used for qPCR.

### Glucose consumption

The treated hBMSCs were harvested and washed twice with PBS. To evaluate the glucose consumption of cells, a glucose uptake kit (colorimetric method) (product number: BC2500, Solarbio, Beijing, China) was used for determination. In the experiment, 2-DG was taken up by glucose transport carriers and then metabolized into 2-deoxyglucose-6-phosphate (2-DG6P). The accumulated 2-DG6P is enzymatically oxidized to produce NADPH, and then, NADPH is specifically detected by a chromogenic NADPH probe. The OD value signal of 570–610 nm was read.

### ATP generation

The treated hBMSCs were harvested and washed twice with PBS. To evaluate the glucose consumption of cells, an ATP content kit (product number: MAK184, Solarbio) was used. Add 50 μL ATP working solution to ATP standard solution, blank control, and test sample in each well to achieve a total ATP measurement volume of 100 μ L/well, stored in dark, let stand at room temperature for 30 min, and absorbance was monitored at an OD value of 570 nm using an absorbance plate reader.

### Lactic acid generation

According to the instructions for using the D-lactate test kit (product number: BC2230, Solarbio), 500 μL of 1% H_2_SO_4_ was added to the fermentation broth, shaken well, mixed, and then centrifuged at 12,000 rpm for 2 min to remove sediment. The optical purity was determined by high-performance liquid chromatography. The detection wavelength was 575 nm.

### Statistical analysis

Cell experiments were performed three times, and all data were analyzed by GraphPad Prism 8.0 (GraphPad Software, San Diego, CA) and expressed as the mean ± standard deviation (SD). Comparisons between two groups were made using *t* tests, which were consistent with a normal distribution. Comparisons between multiple groups were performed using ANOVA with the test conforming to a normal distribution, followed by Fisher’s LSD test to assess differences between groups. *p* < 0.05 was considered statistically significant.

## Results

### ASCL1 increases during osteogenic differentiation in hBMSCs

**Figure 1.**
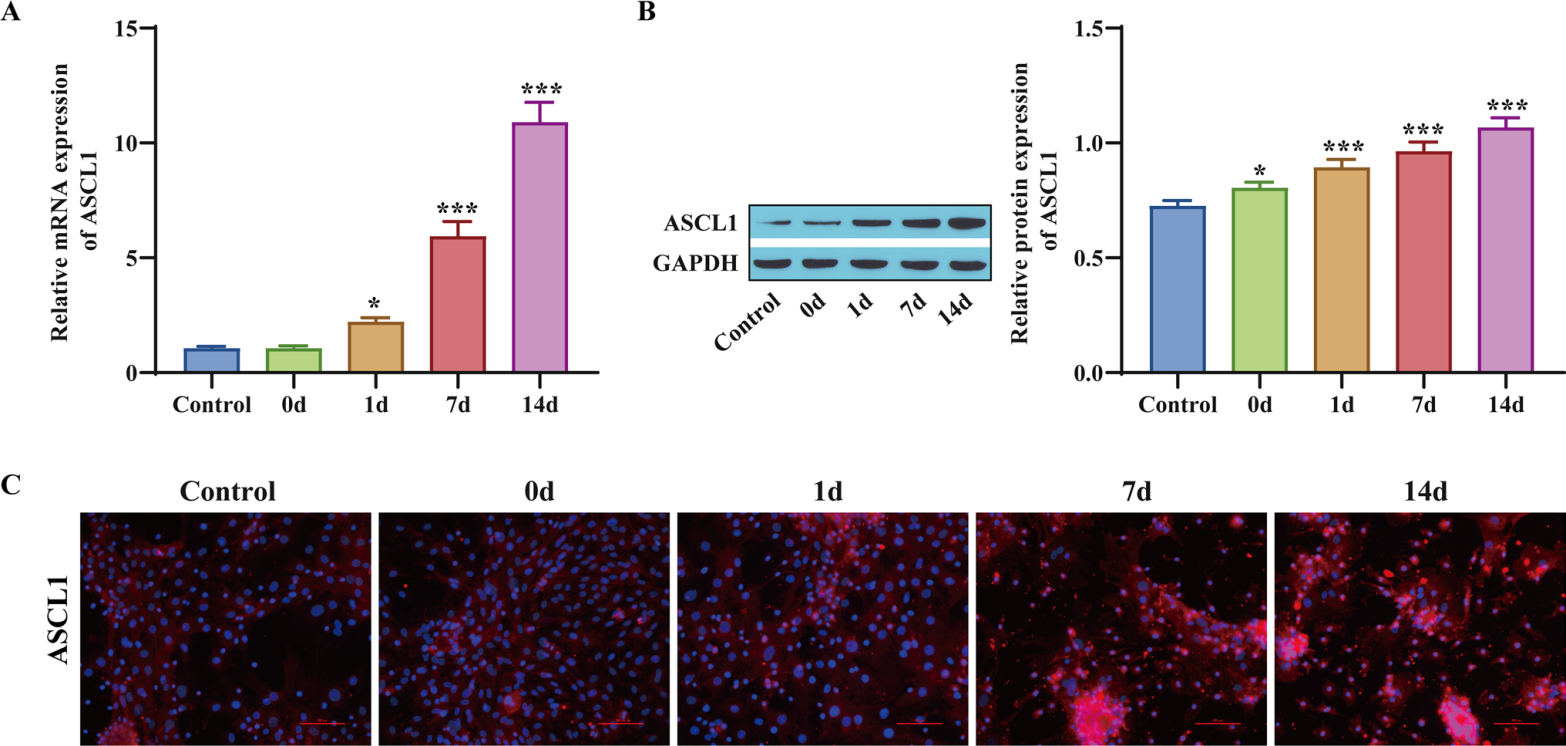
ASCL1 increases during osteogenic differentiation in hBMSCs. (*A*) The expression level of ASCL1 was measured by RT‒qPCR. (*B*) Western blot detection of the level of ASCL1 protein. (*C*) Immunofluorescence detection of ASCL1 levels. *p < 0.05, ***p < 0.001, compared with the Control group.

To determine the expression changes of ASCL1, RT‒qPCR was used to measure ASCL1 levels. The expression level of ASCL1 was significantly upregulated 7 d after hBMSC osteogenic medium induction and upregulated over time (Fig. 1*A*). The protein expression test and immunofluorescence test showed that ASCL1 level significantly increased after 7 d of induction in osteogenic medium and was upregulated with increasing culture time (Fig. 1*B*, *C*). The above results confirm that ASCL1 significantly increases during the osteogenic differentiation process of hBMSCs.

### Overexpression of ASCL1 enhances hBMSC osteogenic differentiation

**Figure 2.**
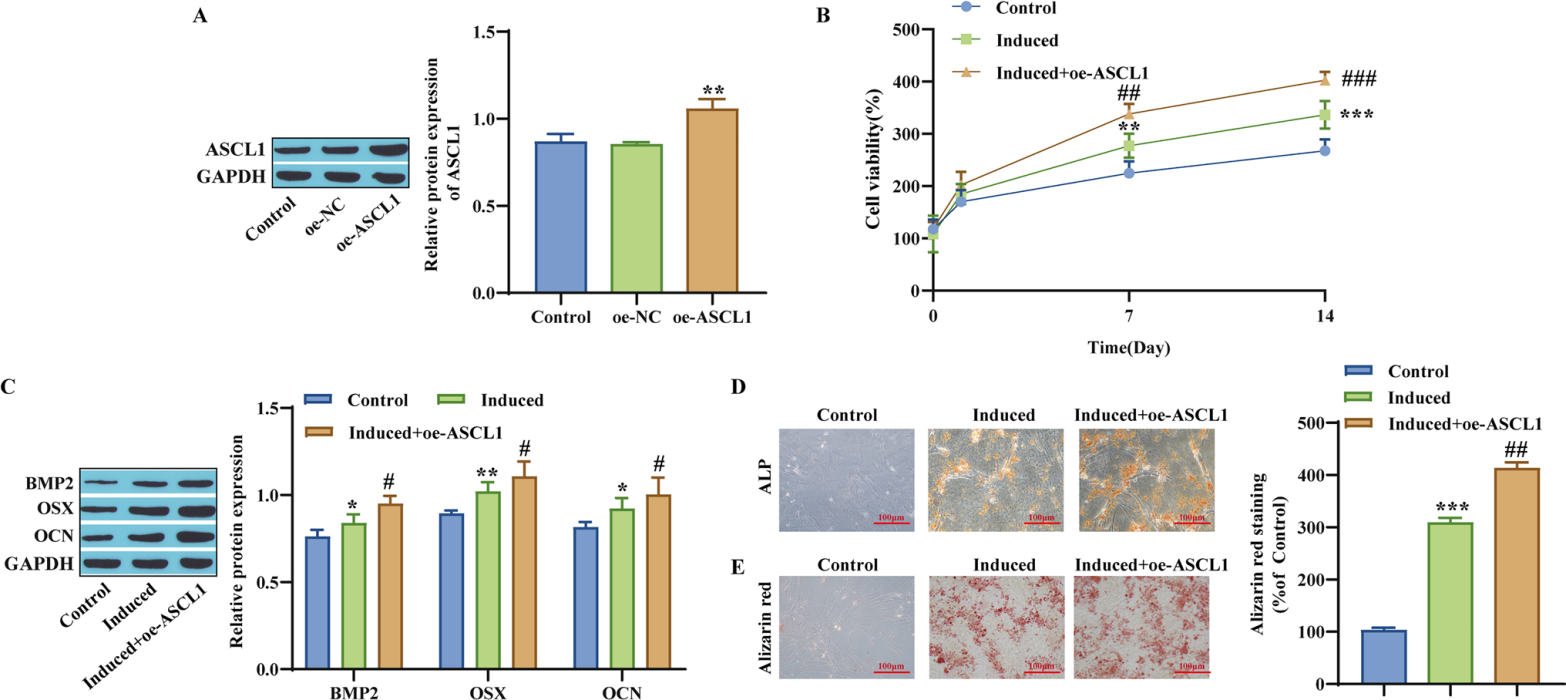
Effect of ASCL1 on the osteogenic differentiation of hBMSCs. (*A*) Western blotting was used to measure the expression level of ASCL1. (*B*) CCK-8 detection of cell proliferation viability. (*C*) The expression levels of BMP2, OSX, and OCN proteins were measured by Western blotting. (*D*) ALP staining. (*E*) Alizarin red staining. *p < 0.05, **p < 0.01, ***p < 0.001, compared with the Control group; ^#^*p* < 0.05, ^##^*p* < 0.001, ^###^*p* < 0.001, compared with the Induced group.

The expression level of ASCL1 was measured after overexpression in hBMSCs. As expected, the expression level of ASCL1 protein was significantly increased after overexpression (Fig. 2*A*). The results of measuring cell proliferation viability showed that the induced group significantly upregulated cell viability after the seventh day of hBMSC osteogenic induction, while the upregulation of cell viability was more pronounced after transfection with oe-ASCL1 (Fig. 2*B*). Western blot detected the expression of osteoblast-related proteins (BMP2, OSX, OCN ), and the results showed that overexpression could further promote the expression of these proteins (Fig. 2*C*). In addition, the degree of osteogenic differentiation of cells after ASCL1 overexpression was also examined, and the presence of yellow precipitates indicated that hBMSCs were differentiating on the seventh day of induced osteogenesis (Induced), and the effect was more significant after oe-ASCL1, promoting significant differentiation participation of hBMSCs (Fig. 2*D*). The experimental results of alizarin red staining also demonstrated that oe-ASCL1 can promote the differentiation process (Fig. 2*E*). In summary, overexpression of ASCL1 enhances the osteogenic differentiation of hBMSCs.

In addition, we also explored the effect of knockdown of ASCL1 on osteogenic differentiation of hBMSC cells and found that the expression level of ASCL1 protein was significantly reduced in the si-ASCL1 group compared with the Control group (Fig. S1A). Cell proliferation viability assay showed that cell viability in the si-ASCL1 group was lower than that in the Induced group (Fig. S1B). Western blotting detected osteogenic differentiation-related proteins OSX, OCN, and BMP2, and the expression of these proteins was reduced by si-ASCL1 (Fig. S1C). And the experimental results of ALP and alizarin red staining also proved that si-ASCL1 inhibited the differentiation process (Fig. S1D-E).

### USP8 ubiquitination regulates ASCL1 expression

**Figure 3.**
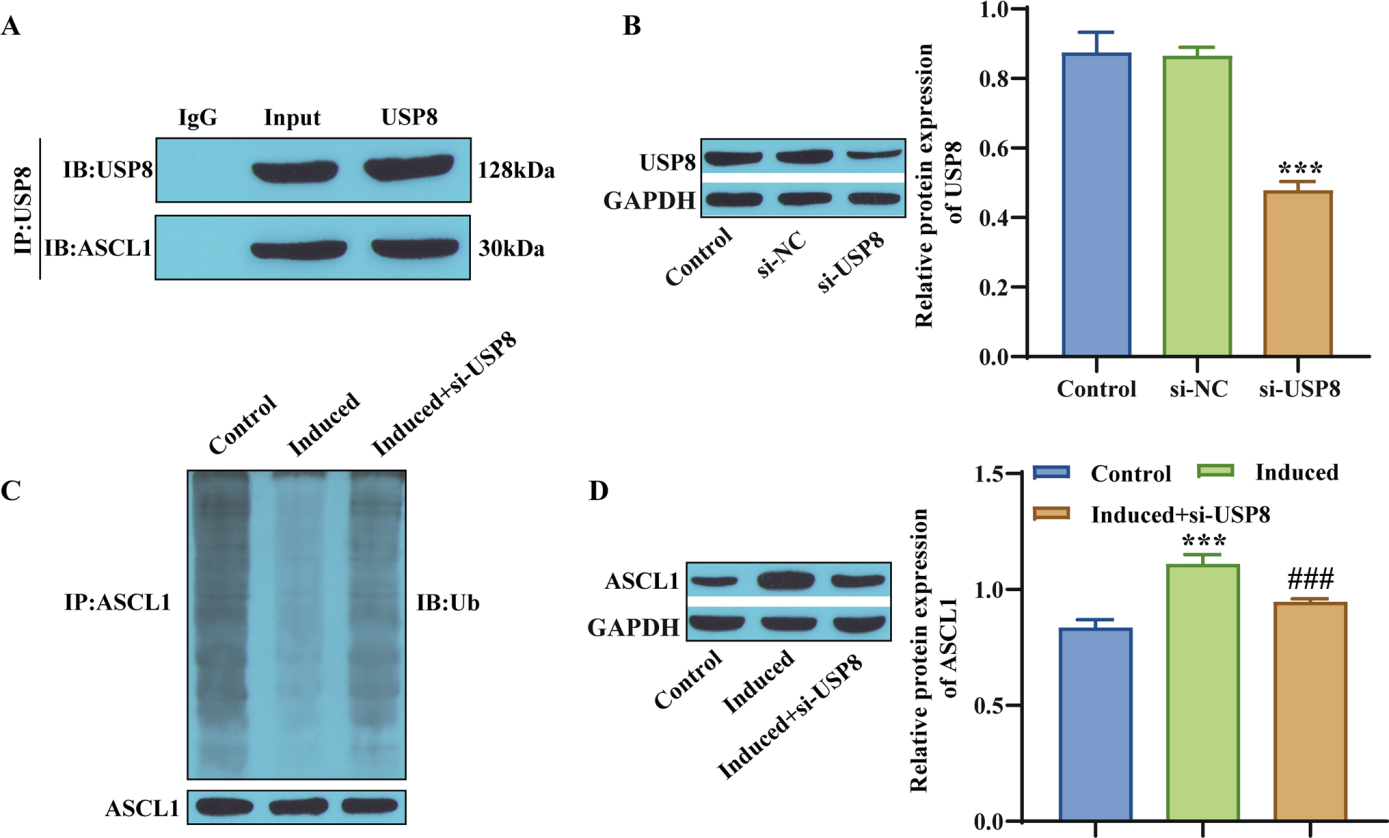
USP8 ubiquitination regulates ASCL1 expression. (*A*) Co-IP determination and analysis of the binding relationship between USP8 and ASCL1. (*B*) Western blot detection of the transfection efficiency of USP8. (*C*) Determination of the ubiquitination level of ASCL1. (*D*) Western blot detection of the ASCL1 protein expression level. ***p < 0.001, compared with the si-NC or Control group; ^###^*p* < 0.001, compared with the Induced group.

USP8 is a key deubiquitinase involved in protein degradation and plays a regulatory role in ASCL1 protein (Huang *et al*. 2022). To further verify the specific regulatory effects, first, Co-IP measurements were conducted, and the results confirmed the interaction between USP8 and ASCL1 (Fig. 3*A*). Next, knockdown of USP8 expression decreased its expression level in hBMSCs (Fig. 3*B*). The ubiquitination level of ASCL1 was measured, and the results showed that knocking down USP8 promoted the occurrence of ASCL1 ubiquitination (Fig. 3*C*). Finally, the decreased expression of USP8 also decreased the expression level of ASCL1 protein (Fig. 3*D*). In summary, USP8 ubiquitination regulates ASCL1 expression.

### ASCL1 upregulates CD47 transcription and promotes hBMSC osteogenic differentiation

**Figure 4.**
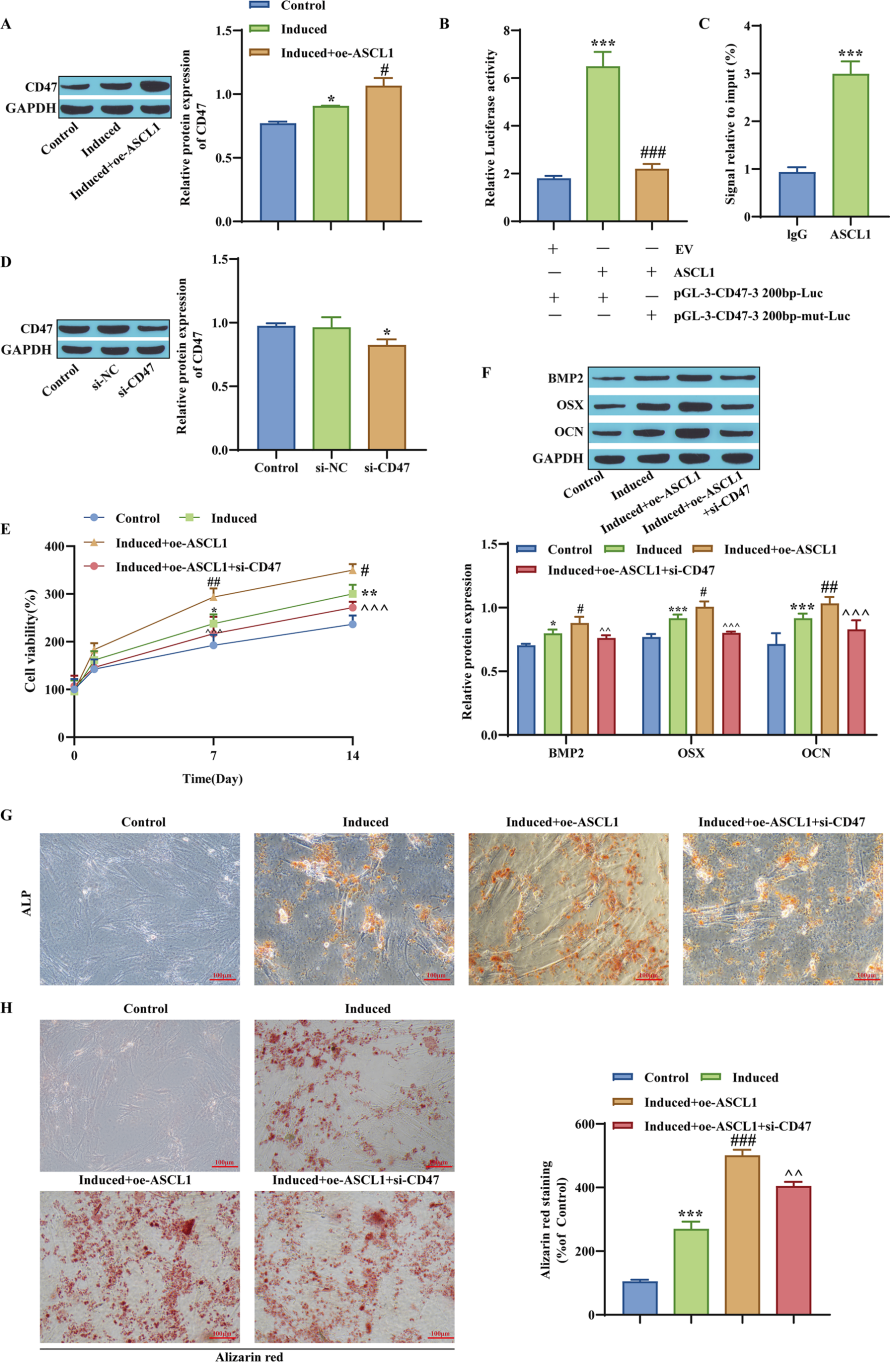
ASCL1 upregulates CD47 transcription and promotes hBMSC osteogenic differentiation. (*A*) Western blot detection of CD47 levels in hBMSCs. (*B*) The binding relationship between ASCL1 and the CD47 promoter was determined by a luciferase promoter assay. (*C*) Determination of the binding ability of ASCL1 and CD47. (*D*) Western blot detection of CD47 transfection efficiency. (*E*) CCK-8 detection of cell proliferation viability. (*F*) Western blot detection of the osteogenic differentiation-related proteins BMP2, OSX, and OCN. (*G*) ALP activity measurement. (*H*) Alizarin red staining. *p < 0.05, **p < 0.01, ***p < 0.001, compared with the Control group; ^#^*p* < 0.05, ^##^*p* < 0.01, ^###^*p* < 0.001, compared with the Induced group; ^^*p* < 0.01, ^^^*p* < 0.001, compared with the Induced + oe-ASCL1 group.

It has been shown that protein stability and nuclear localization of ASCL47 can activate CD47 transcription (Wang *et al*. 2022a). Next, we will further explore the effect of ASCL1-mediated CD47 transcription on hBMSC osteogenic differentiation. After Western blot detection, it was found that the expression of CD47 in the induced group was upregulated, while overexpression of ASCL1 further enhanced the expression of CD47 (Fig. 4*A*). The luciferase promoter determined the binding relationship between ASCL1 and the CD47 promoter (Fig. 4*B*). To check whether ASCL1 can directly bind to the CD47 promoter, a ChIP qPCR assay was performed. After adding ASCL1, the promoter activity of CD47 was upregulated, which confirms the combination theory (Fig. 4*C*). To further verify whether ASCL1 can serve as an upstream factor regulating CD47 expression, CD47-specific siRNA was knocked down, and the expression level of CD47 was significantly downregulated after knockdown of CD47 (Fig. 4*D*), indicating the success of si-CD47. In addition, transfection with oe-ASCL1 enhanced cell proliferation viability, while knocking down CD47 in the Induced + oe-ASCL1 group decreased cell viability (Fig. 4*E*). The detection of osteogenic differentiation-related proteins showed that overexpression of ASCL1 enhanced the osteogenic differentiation of hBMSCs, while si-CD47 reversed this phenomenon (Fig. 4*F*). It was also found in subsequent osteogenic analysis experiments that overexpression of ASCL1 further promoted osteogenic differentiation of hBMSCs. After si-CD47 treatment, the positive effect of ASCL1 overexpression on the osteogenic differentiation of hBMSCs was weakened (Fig. 4*G*), and the same results were observed by alizarin red staining (Fig. 4*H*). In conclusion, ASCL1 regulates CD47 transcription and promotes hBMSC differentiation.

### CD47 activates the AKT signaling pathway, thereby promoting hBMSC glycolysis

**Figure 5.**
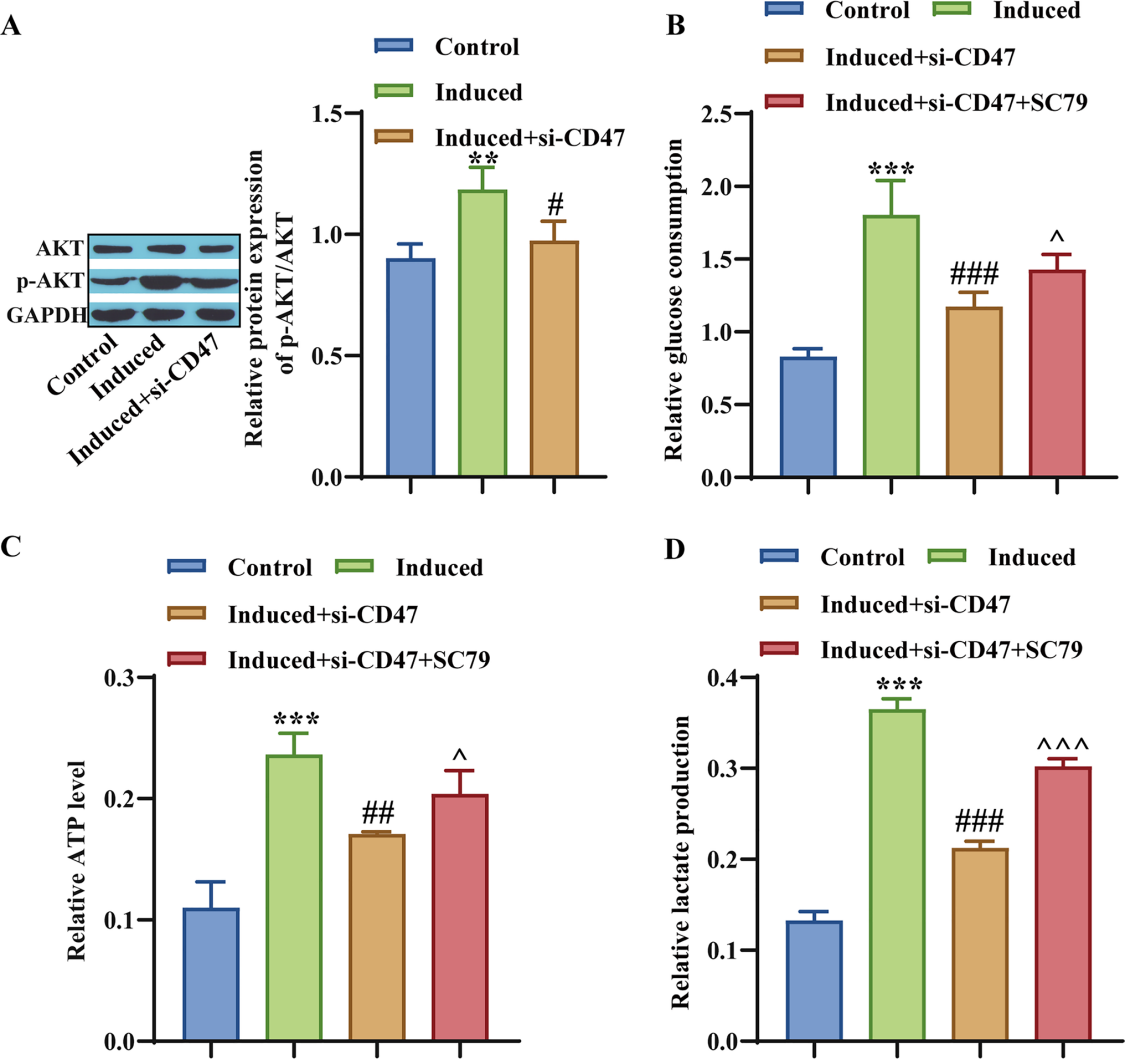
Effect of CD47 on the AKT signaling pathway and glycolysis in hBMSCs. (*A*) Western blot detection of AKT and p-AKT proteins. (*B*) Determination of glucose consumption level. (*C*) Determination of ATP generation using a reagent kit. (*D*) The reagent kit determines the generation of lactic acid. **p < 0.01, ***p < 0.001, compared with the Control group; ^#^*p* < 0.05, ^##^*p* < 0.01, ^###^*p* < 0.001, compared with the Induced group; ^*p* < 0.05, ^^^*p* < 0.001, compared with the Induced + si-CD47 group.

Akt signaling can influence the osteogenic differentiation of MSCs, while the AKT signaling pathway induces osteogenic differentiation and angiogenesis (Hu *et al*. 2022). Next, our study explored the effect of CD47 on the AKT signaling pathway, which promotes hBMSC glycolysis. First, the phosphorylation level of AKT in cells transfected with si-CD47 was significantly lower than that in the induction group (Fig. 5*A*). Subsequently, we measured intracellular glucose consumption levels, ATP production, and lactate production levels. Compared with the induced group, after transfection with si-CD47, intracellular glucose consumption levels, ATP production, and lactate production levels were significantly reduced, while the AKT activator (SC79) reversed this effect (Fig. 5*B*–*D*). The above results confirm that CD47 activates the AKT signaling pathway, thereby promoting glycolysis in hBMSCs.

### Ubiquitination of ASCL1 mediates CD47 transcription to activate the AKT signaling pathway and increases hBMSC glycolysis to promote osteogenic differentiation

**Figure 6.**
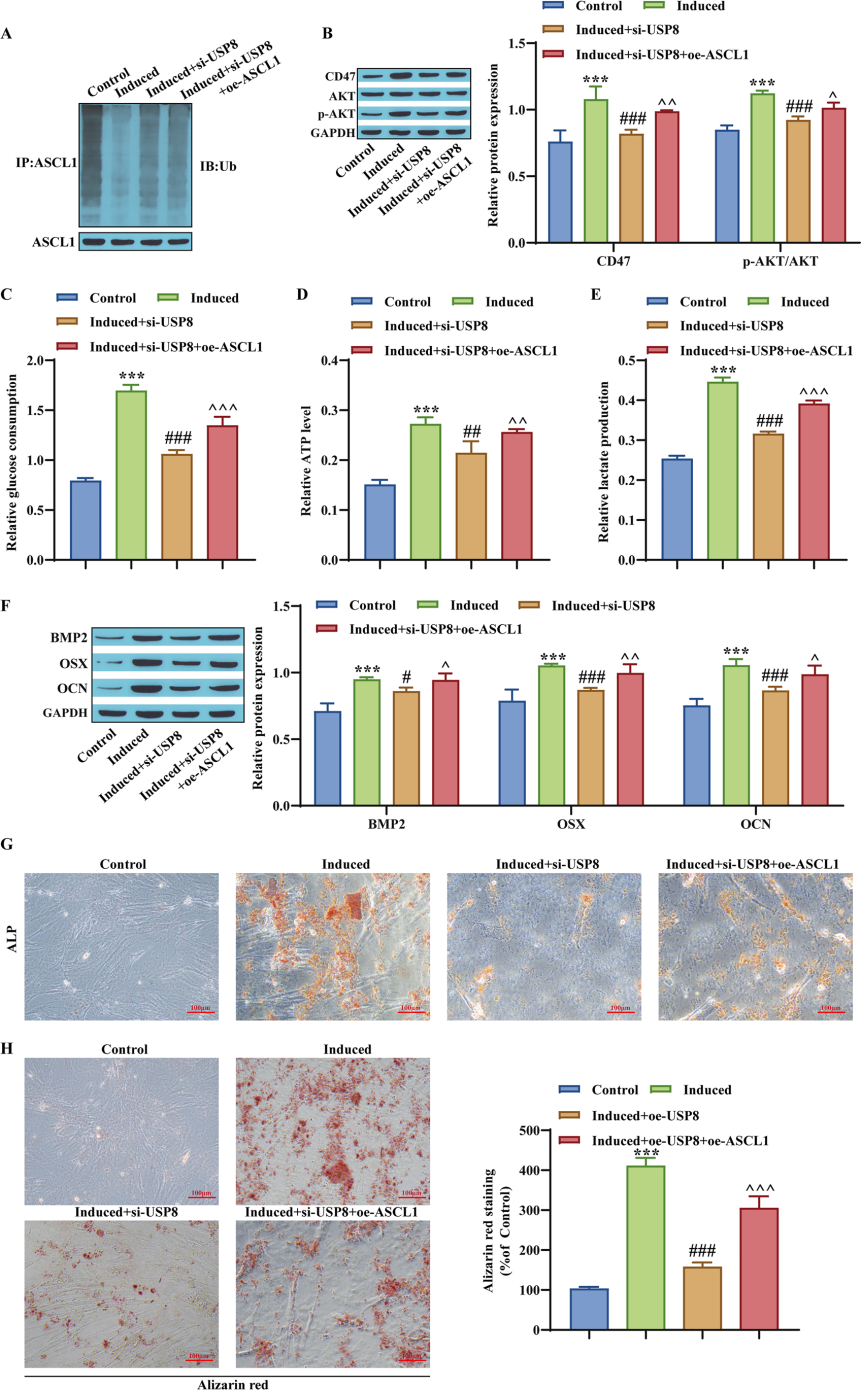
Ubiquitination of ASCL1 mediates CD47 transcription to activate the AKT signaling pathway, increasing hBMSC glycolysis to promote osteogenic differentiation. (*A*) ASCL1 ubiquitination level determination. (*B*) Western blot detection of CD47, AKT, and p-AKT expression levels. (*C*) Determination of glucose consumption level. (*D*) Determination of ATP formation. (*E*) Determination of lactate production using a reagent kit. (*F*) Western blot detection of osteogenic differentiation-related proteins. (*G*, *H*) Differentiation activity of hBMSCs and detection of mineralized water level. ***p < 0.001, compared with the Control group; ^#^*p* < 0.05, ^##^*p* < 0.01, ^###^*p* < 0.001, compared with the induced group; ^*p* < 0.05, ^^*p* < 0.01, ^^^*p* < 0.001, compared with the Induced + si-USP8 group.

Then, a final experiment was conducted to elucidate the molecular mechanism. Western blot detection of ASCL1 ubiquitination and CD47 expression levels, AKT, and p-AKT. The results showed that knocking down USP8 significantly upregulated ASCL1 ubiquitination levels in the induced group, while CD47 and AKT phosphorylation levels were downregulated. Later, oe-ASCL1 alleviated the downregulation of CD47 and AKT phosphorylation levels, while ASCL1 ubiquitination levels remained unchanged (Fig. 6*A*, *B*). Next, glucose consumption levels, ATP production, and lactate production levels were measured. Knocking down USP8 resulted in a decrease in glucose consumption, ATP production, and lactate production. Then, oe-ASCL1 reversed this expression effect (Fig. 6*C*–*E*). Western blot analysis showed that knocking down USP8 reduced the expression levels of BMP2, OSX, and OCN proteins. Later, oe-ASCL1 alleviated this expression effect (Fig. 6*F*). ALP measurement and alizarin red staining showed an increase in cell differentiation after knocking down USP8, while overexpression of ASCL1 alleviated the process of cell differentiation (Fig. 6*G*, *H*). The above results indicate that the ubiquitination of ASCL1 mediates CD47 transcription to activate the AKT signaling pathway and increase hBMSC glycolysis to promote osteogenic differentiation.

## Discussion

Osteoporosis is a common bone metabolic disease that leads to bone loss and fracture risk and is commonly seen in elderly and postmenopausal women (Yong and Logan 2021; Zhang *et al*. 2022). The imbalance between bone resorption and bone formation caused by reduced osteoblast activity is the main cause of the inability to maintain bone homeostasis (McNamara 2021). BMSCs are an important precursor of osteoblasts and a major source of osteoclasts (Guo *et al*. 2021), and elucidating the regulatory mechanisms of osteogenic differentiation of hBMSCs is crucial for identifying new therapeutic targets for osteoporosis.

ASCL1 is a member of the basic helix-loop-helix (bHLH-like) family of transcription factors, a family of bHLH-like proteins with structural domains that bind specifically to the E-BOX (CACCTG) promoter, which can dominate the cellular transformation toward neural direction (Baronti *et al*. 2017). The link between ASCL1 and the development of stem cell and neuronal phenotypes was confirmed, for example, in studies targeting prostate cancer (Nouruzi *et al*. 2022). However, few studies have reported the role of ASCL1 in hBMSCs. The human ASCL1 gene is located on the long arm of chromosome 12, 12q23.2, and its coding region is approximately 711 bp in length. The coding product of human ASCL1 gene is a peptide chain consisting of 236 amino acids with a molecular weight of approximately 34 kDa. In this study, we found that ASCL1 protein was significantly enhanced in hBMSCs after 7 d of induction, and overexpression of ASCL1 significantly increased the proliferative viability, ALP activity, and mineralized nodules of hBMSCs and promoted the osteogenic differentiation of hBMSCs. ALP is one of the most reliable markers of osteogenic differentiation; it is expressed earlier in osteoblasts, and ALP activation is required for bone mineralization (Long *et al*. 2023b). Meanwhile, in a study of Cushing’s disease, ASCL1 was found to be overexpressed in USP8 mutant and wild-type tumors (Chen *et al*. 2022). USP8 is a cysteine ubiquitinase belonging to the USP family, and a large number of studies have reported on the role of USP8-mediated ubiquitination in cancer (Peng *et al*. 2020; Xie *et al*. 2022). In this study, we confirmed the interaction between USP8 and ASCL1 by Co-IP results and increased ubiquitination of ASCL1 after knockdown of USP8.

Previous studies have found that CD47 is involved in osteoblast and stromal cell/osteoblast differentiation in mouse bone marrow cultures and that a lack of CD47 severely impairs SIRPα-dependent osteoblast differentiation and leads to a reduction in osteoclast formation (Koskinen *et al*. 2013). In a report by Wang *et al*. studying small cell lung cancer, it was found that increased protein stability and nuclear localization of ASCL1 activated CD47 transcription, which enhanced cancer stem cell properties and evaded phagocytosis in small cell lung cancer (Wang *et al*. 2022a). By searching the JASPAR database (http://jaspar.genereg.net/), we found that ASCL1-binding motif mutations reduced ASCL1-mediated CD47 promoter activity, and the binding of ASCL1 to the promoter region of the CD47 gene in hBMSCs was confirmed using ChIP‒qPCR. To further verify whether ASCL1 could act as an upstream factor regulating CD47 expression, si-CD47 was transfected into hBMSCs, and the results showed that transfection of si-CD47 inhibited the promotion of osteogenic differentiation of hBMSCs by ASCL1.

Previous studies have shown that CD47 can act as a predictor of various cancers, affecting cell proliferation, migration, and apoptosis in various malignancies through the PI3K/Akt pathway (Liu *et al*. 2019). Akt inhibits the target of mammalian target of rapamycin (mTOR) in gliomas, which regulates a variety of cellular responses, such as cytoskeletal structure, transcription, and autophagy, to enhance cell viability (Revathidevi and Munirajan 2019). Phosphorylated Akt (p-Akt) is thought to induce signals that interfere with apoptosis and promote cell proliferation and motility through the activation of mTOR, an important mechanism (Cao *et al*. 2017). In our study, the reduction in AKT phosphorylation after knockdown of CD47 in hBMSCs was also evident. In addition, AKT signaling is involved in the cellular glycolytic pathway, leading to chemoresistance in cancer cells (Zhang *et al*. 2021; Tantai *et al*. 2022). Glycolysis plays an important regulatory role in various diseases, and osteoblasts metabolize glucose to lactate via aerobic glycolysis during osteogenic differentiation, which promotes osteoblastic differentiation (Nian *et al*. 2022). Wang *et al*. (2022b) demonstrated that inhibition of PFKFB3-mediated glycolysis significantly attenuated human valvular mesenchymal cell (hVIC) osteogenic differentiation and inflammation. In the present study, our study confirmed that after transfection with si-CD47, hBMSCs showed reduced levels of glucose consumption and decreased levels of ATP production, lactate production, ALP activity, and mineralized nodules.

In summary, this study indicates that USP8 ubiquitination regulates ASCL1 expression and mediates CD47 transcriptional activation of the AKT signaling pathway and that glycolysis promotes osteogenic differentiation of hBMSCs. This study provides evidence that elucidates the potential regulatory mechanisms of ASCL1 in the development of osteogenic differentiation in vitro and establishes a theoretical basis for improving osteoporosis.

### Supplementary Information

Below is the link to the electronic supplementary material.

Fig. S1 Effect of knockdown ASCL1 on the osteogenic differentiation of hBMSCs. *A* Western blotting was used to measure the expression level of ASCL1; *B* CCK-8 detection of cell proliferation viability; *C* The expression levels of BMP2, OSX and OCN proteins were measured by Western blotting; *D* ALP staining; *E* Alizarin red staining. ** p<0.01, *** p<0.001, compared with the Control group; ^#^p<0.05, ^##^p<0.001, ^###^p<0.001, compared with the Induced group.(PNG 1.4 mb)

High resolution image (TIF 4.25 mb)

## Data Availability

The datasets used and/or analyzed during the current study are available from the corresponding author upon reasonable request.
