# Supplementary figures and images for: Ubiquitination of ASCL1 mediates CD47 transcriptional activation of the AKT signaling pathway, and glycolysis promotes osteogenic differentiation of hBMSCs
Source: In Vitro Cell Dev Biol Anim. 2023 Oct 2;59(8):636–48. doi: 10.1007/s11626-023-00811-0 (PMC10567835; doi:10.1007/s11626-023-00811-0)

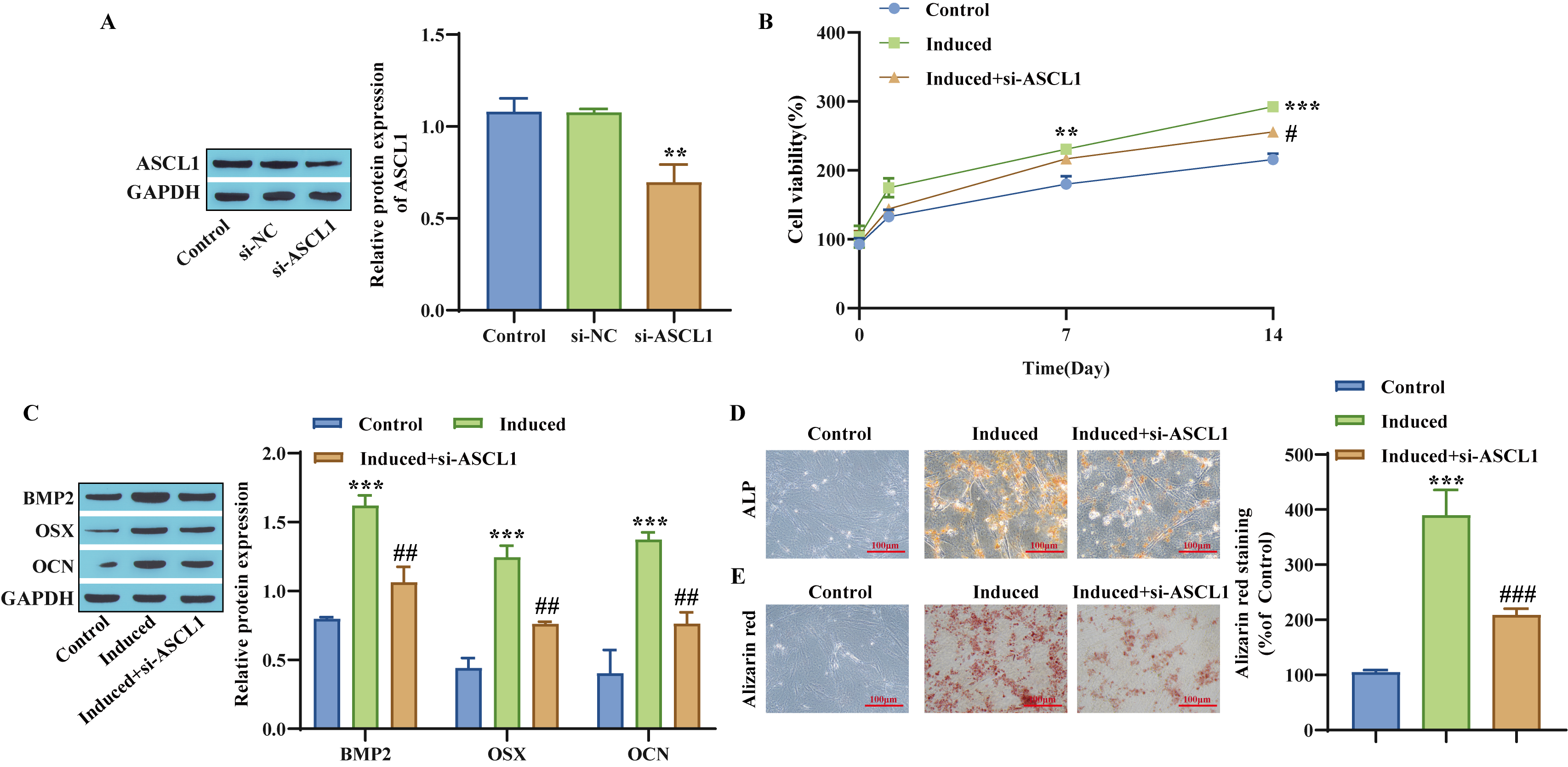

Supplement: Supplementary file 1 — Effect of knockdown ASCL1 on the osteogenic differentiation of hBMSCs. A Western blotting was used to measure the expression level of ASCL1; B CCK-8 detection of cell proliferation viability; C The expression levels of BMP2, OSX and OCN proteins were measured by Western blotting; D ALP staining; E Alizarin red staining. ** p<0.01, *** p<0.001, compared with the Control group; #p<0.05, ##p<0.001, ###p<0.001, compared with the Induced group.(PNG 1.4 mb) [file 11626_2023_811_Fig7_ESM.png]

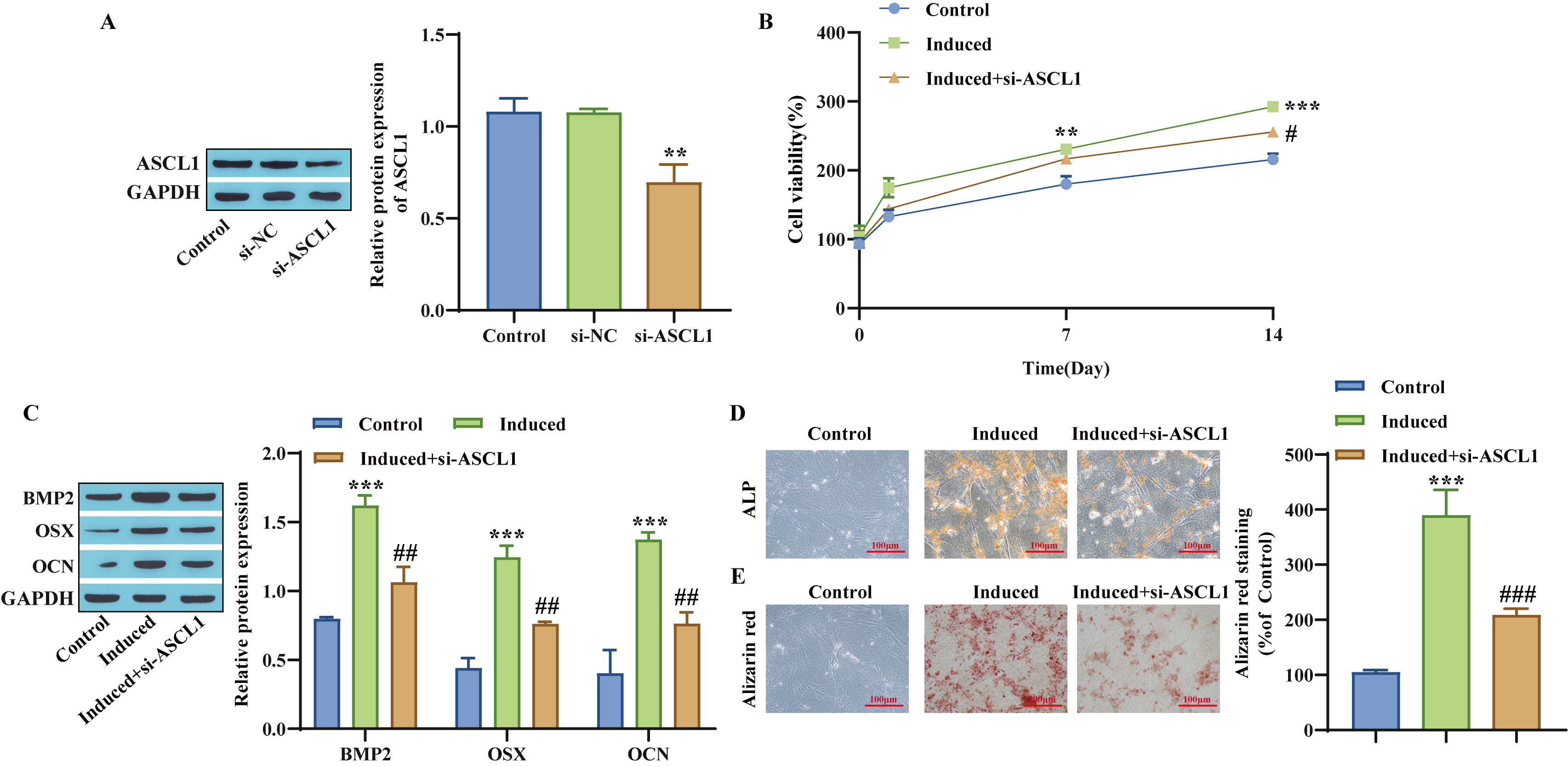

Supplement: Supplementary file 2 — High resolution image (TIF 4.25 mb) [file 11626_2023_811_MOESM1_ESM.tif]
